# Albumin-muscle density score predicts overall survival in patients with hepatocellular cancer undergoing treatment with transarterial chemoembolization

**DOI:** 10.1007/s00432-024-06043-3

**Published:** 2024-11-30

**Authors:** Alexey Surov, Andreas Wienke, Jan Borggrefe, Timo Alexander Auer, Bernhard Gebauer, Aline Mähringer-Kunz, Felix Nensa, Johannes Haubold, Benedikt Michael Schaarschmidt, René Hosch, Jens Kleesiek, Thierno D. Diallo, Natascha Roehlen, Dominik Bettinger, Michel Eisenblätter, Verena Steinle, Philipp Mayer, David Zopfs, Daniel Pinto dos Santos, Lukas Müller, Roman Kloeckner

**Affiliations:** 1 Department of Radiology, Neuroradiology and Nuclear Medicine, Johannes Wesling University Hospital Minden, Ruhr University Bochum, Bochum, Germany; 2 Department of Radiology, Charité - University Medicine Berlin, Berlin, Germany; 3 Institute of Medical Epidemiology, Biostatistics, and Informatics, Martin-Luther- University Halle-Wittenberg, Halle, Germany; 4 Department of Diagnostic and Interventional Radiology, University Medical Center of the Johannes Gutenberg University Mainz, Langenbeckst. 1, 55131 Mainz, Germany; 5 Department of Diagnostic and Interventional Radiology and Neuroradiology, University Hospital Essen, Essen, Germany; 6 Department of Diagnostic and Interventional Radiology, Freiburg University Hospital, Freiburg, Germany; 7 Department of Diagnostic and Interventional Radiology, Medical Faculty OWL, Bielefeld University, Bielefeld, Germany; 8 Department of Diagnostic and Interventional Radiology, University Medical Center Heidelberg, Heidelberg, Germany; 9 Department of Radiology, University Hospital Cologne, Cologne, Germany; 10 Department for Interventional Radiology, University Hospital of Lübeck, Ratzeburger Allee 160, Lübeck, Germany

**Keywords:** Hepatocellular cancer, Skeletal musculature, Survival, Transarterial chemoembolization

## Abstract

**Purpose:**

The purpose of the present study was to analyze associations between different skeletal muscle quality parameters and survival in patients with hepatocellular carcinoma (HCC) undergoing treatment with transarterial chemoembolization (TACE).

**Methods:**

We retrospectively enrolled 784 treatment-naïve patients with HCC undergoing TACE at six tertiary care centers between 2010 and 2020. Intramuscular adipose tissue (IMAT) and skeletal muscle density (SMD) were estimated. Myosteatosis was defined as SMD < 28.0 HU for men and < 23.8 HU for women. Furthermore, albumin-SMD score (ADS) was calculated as follows: serum albumin (g/dL) × SMD (HU). To assess the impact of muscle quality on survival, Cox regression model was used. Kaplan-Meier curves were used for survival analysis. Parameters of skeletal muscle quality were compared in univariate and multivariate regression analyses, adjusted for established risk factors.

**Results:**

In the overall sample, survivors had higher SMD and ADS in comparison to non-survivors. Patients with low ADS had a lower OS than patients with high ADS (8.4 vs. 14.3 months, *p* < 0.001). In alcohol-induced HCC, none of the analyzed parameters of muscle quality influenced survival. In viral induced HCC, patients with low ADS had lower OS than patients with high ADS (8.8 vs. 15.7 months, *p* < 0.001). In patients with non-alcoholic steatohepatitis (NASH), none of the analyzed parameters of muscle quality influenced survival.

**Conclusions:**

Low ADS is an independent predictor of worse OS in patients with viral-induced HCC undergoing treatment with TACE. In alcohol-induced and NASH-induced HCCs, parameters of muscle quality do not influence OS.

**Supplementary Information:**

The online version contains supplementary material available at 10.1007/s00432-024-06043-3.

## Key points

• In alcohol-induced HCC, parameters of skeletal muscle quality do not predict OS.
• In viral-induced HCC, low albumin-density score is an independent and strong predictor of worse OS.
• In NASH/NAFLD induced HCC, low albumin-density scoe is associated with worse OS.

**Supplementary Information:**

The online version contains supplementary material available at 10.1007/s00432-024-06043-3.

## Introduction

Hepatocellular carcinoma (HCC) is the most common primary liver cancer and one of the most common causes of cancer-related mortality worldwide (Sung et al. 2021).

Cross-sectional imaging like computed tomography (CT) and magnetic resonance imaging (MRI) play an essential role in diagnosis and staging of HCC (Galle et al. 2018). Moreover, both CT and MRI parameters, such as intensity and/or homogeneity of contrast medium enhancement can also provide data about tumor prognosis. For instance, patients with ill-defined tumor margins had worse survival compared to patients with well-defined tumor margins (Mukund et al. 2021). Also peritumoral hypointensity of HCC on MRI in hepatobiliary phase was reported as another imaging biomarker for worse survival and increased tumor recurrence risk (Öcal et al. 2023).

Furthermore, cross-sectional imaging can also provide data about patient`s condition. There is evidence in the literature that the status of the skeletal musculature plays a prognostic role in oncology (Surov et al. 2023). So far, low skeletal muscle mass (LSMM) on CT predicts overall survival in patients with HCC both in the curative and in the palliative setting (March et al. 2022). Also, LSMM predicts treatment response and therapy-associated toxicity in HCC (Surov et al. 2021; Mir et al. 2012).

Recently, it has been shown that myosteatosis or fatty infiltration of the skeletal musculature plays a greater prognostic role in oncology than LSMM (Aleixo et al. 2020; Hamaguchi et al. 2015). Some studies also analyzed the prognostic role of myosteatosis in HCC (Fujiwara et al. 2015; Kaibori et al. 2015; Labeur et al. 2019). However, the reported results are conflicting. For instance, Fujiwara et al. reported that myosteatosis is able to predict overall survival in patients with HCC (Fujiwara et al. 2015). Similar results were reported by Kaibori et al. (Kaibori et al. 2015). In the study of Labeur et al., there were no associations between myosteatosis and survival (Labeur et al. 2019). Furthermore, most reports analyzed relationships between myosteatosis and clinical outcomes in a curative setting, i.e. in patients undergoing surgical resection of HCC. In patients with intermediate-stage HCC undergoing treatment with transarterial chemoembolization (TACE), the clinical impact of the skeletal muscle quality is still unclear.

Therefore, the purpose of the present study was to analyze the prognostic role of different skeletal muscle quality parameters in patients with HCC, who were treated with TACE.

## Materials and methods

This multicentric retrospective study was approved by the Ethics committee of the Medical Association of Rhineland Palatinate, Mainz, Germany (permit number 2021–15913). The other responsible Ethics committees followed this approval.

### Patients

The present work is a sub-analysis of a multicenter retrospective study (Müller et al. 2022). For this study, data from six German tertiary care centers were collected. The inclusion criteria were: (1) TACE performed between January 2010 and December 2020; (2) age > 18 years; (3) a histological- or image-derived HCC diagnosis, based on EASL (European Association for the Study of Liver Disease) criteria; (4) no treatment performed prior to TACE; (5) no liver transplantation or tumor resection performed during the follow-up period after TACE; and (6) computed tomography (CT) performed prior to treatment initiation. The exclusion criteria were: (1) any treatment performed prior to TACE; (2) liver transplantation or tumor resection during the follow-up period after TACE; (3) missing CT images prior to treatment initiation; (4) insufficient image quality (Müller et al. 2022).

Overall, 784 patients were included. There were 139 women (17.7%) and 645 men (82.3%) with a mean age of 66.4 ± 9.5 years. Patient and tumor characteristics are given in Table 1. The following baseline characteristics were collected for the study: demographic data, liver disease status and etiology, laboratory parameters including albumin and bilirubin levels, and tumor growth pattern, number of lesions, and the diameter of the largest target lesion.

**Table 1:** Baseline characteristics of the included patients

Variable	All patients (*n* = 784)
Age in years, median (IQR)	67 (60–74)
Sex, *n* (%)	
Female	139 (17.7)
Male	645 (82.3)
Etiology, *n* (%)	
alcohol	333 (42.5)
viral	229 (29.2)
NASH	42 (5.4)
alcohol + viral	19 (2.4)
autoimmune hepatitis	8 (1.0)
Hemochromatosis	13 (1.7)
alpha-1 antitrypsin deficiency	1 (0.1)
cardiac cirrhosis	1 (0.1)
cryptogenic	138 (17.6)
Child-Pugh stage, *n* (%)	
No cirrhosis	82 (10.4)
A	394 (50.3)
B	260 (33.2)
C	48 (6.1)
BCLC stage, *n* (%)	
0	10 (1.3)
A	219 (27.9)
B	388 (49.5)
C	137 (17.5)
D	30 (3.8)
Size of the largest lesion in mm, median (IQR)	40.5 (27.0–60.0)
Tumor burden score (TBS), median	5.1 (1.3–24.9)
Number of lesions, median (IQR)	2 (1–3)
Albumin level, g/L, median (IQR)	35 (30–39)

BCLC, Barcelona Clinic Liver Cancer

NASH, non-alcoholic steatohepatitis

HCC was staged using the Barcelona Clinic Liver Cancer (BCLC) classification. Diagnosis of HCC was made according to the current guidelines mainly by imaging (CT or dynamic contrast enhanced MRI). In all patients transarterial interventions were performed by experienced board-certified interventional radiologists. Based on the individual decision of the interventionalist, 56.4% of the patients received drug-eluting bead-TACE and 43.6% conventional TACE.

Furthermore, tumor burden score (TBS) was calculated as follows: TBS = square root ((maximum tumor diameter)^2^ + (number of tumors)^2^) (Müller et al. 2022). We used the cut off value of 3.36 to discriminate patients with low and moderate/high TBS (Mukund et al. 2021).

### Analysis of skeletal muscle quality

**Fig. 1:**
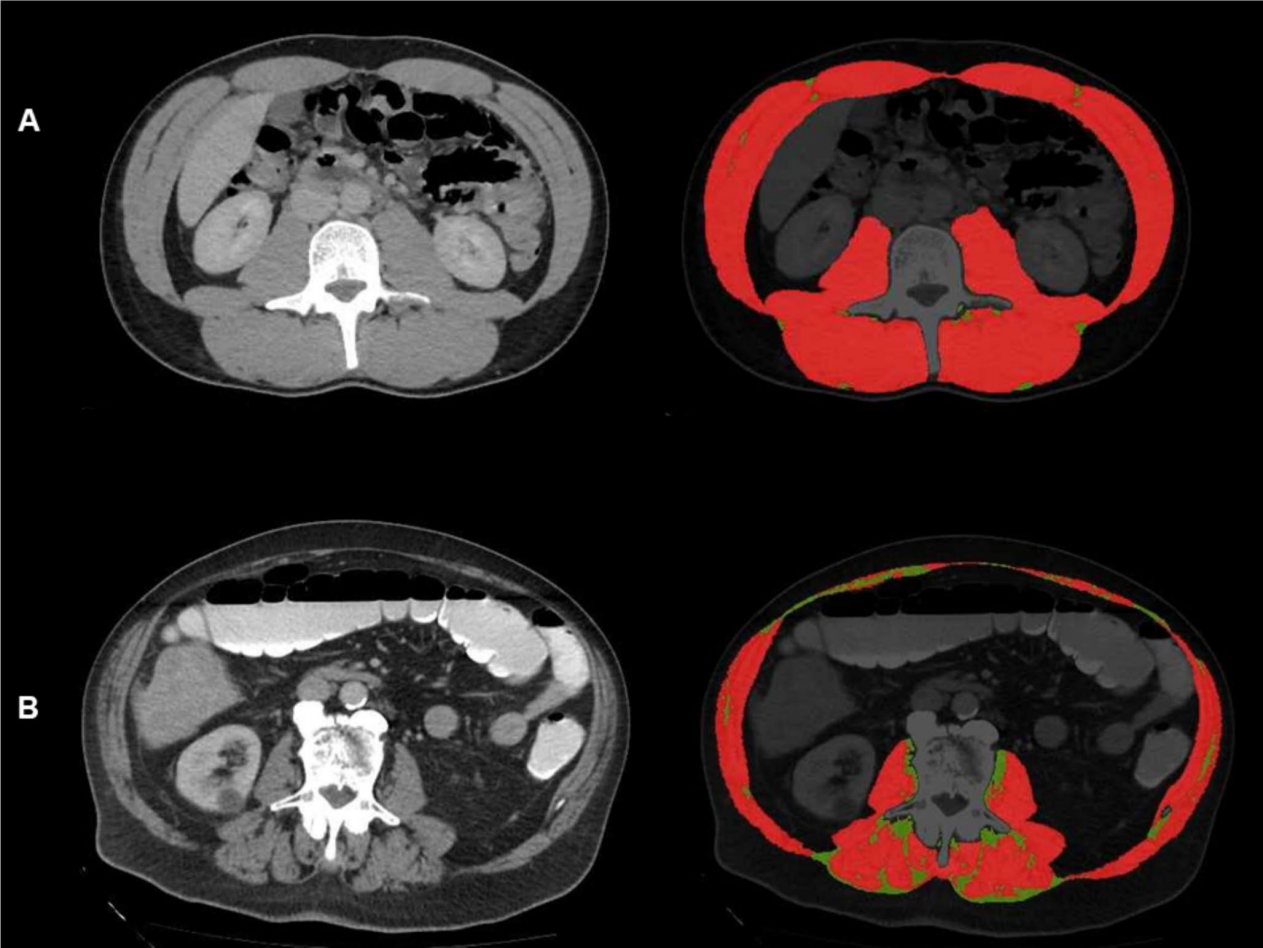
Representative cases of the patient sample with performed segmentation of the skeletal musculature (red) and intramuscular adipose tissue (green). **a**. Patient with high muscle quality (high muscle density and low intramuscular adipose tissue). **b**. Patient with low muscle quality (low muscle density and high intramuscular adipose tissue)

In every case, CT scans (soft tissue window, portal venous phase, and scan thickness of 3–5 mm) at baseline before TACE were used (Suppl. Table 1) The CT scans were collected and transferred to our body composition tool (Fig. 1) (Haubold et al. 2024). Thereafter, a segmentation of the skeletal musculature was performed by using the threshold values of − 29 and 150 HU (Haubold et al. 2024). The following parameters of muscle quality were estimated: skeletal muscle density (SMD) and intramuscular adipose tissue (IMAT). Low skeletal muscle density was defined as skeletal muscle density (SMD) < 28.0 HU for men and < 23.8 HU for women, using the thresholds defined by Sjøblom et al. (Sjøblom et al. 2016). Sex-dependent median value was used as threshold for IMAT in our sample. Furthermore, albumin-density score (ADS) was calculated as follows: serum albumin (g/dL) × SMD (HU) (Kim et al. 2023). Also for ADS, sex-dependent median values were used as cut offs.

### Statistical analysis

For statistical analysis SPSS (version 28, IBM SPSS Statistics for Windows, Armonk, NY, USA: IBM corporation) was used. Collected data (convenience sample) were evaluated by means of descriptive statistics such as absolute and relative frequencies for categorical variables and means and standard deviations as well as medians and interquartile ranges (IQR) for continuous variables. Groups were compared by chi-square test for binary outcomes and by t-test for continuous outcomes. Kaplan-Meier curves were used for the analysis of overall survival (OS). The influence of variables on OS was evaluated by Cox regression. The resulting p-values were interpreted in an exploratory sense.

## Results

**Fig. 2:**
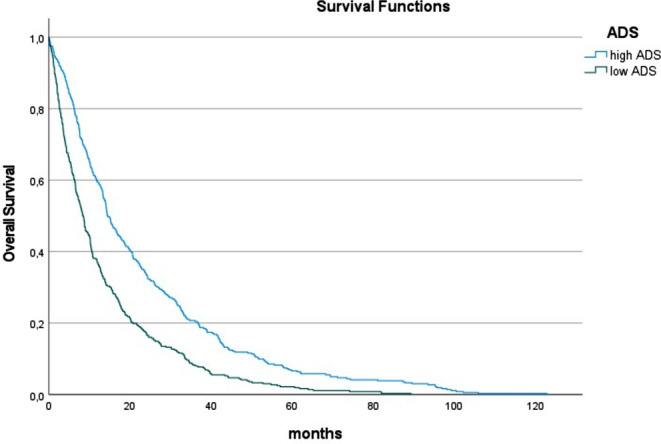
Overall survival in patients with low and normal albumin x muscle density scores (ADS) in the overall sample. Patients with low ADS had lower median OS time in comparison to patients with high ADS (8.4 vs. 14.3 months, *p* < 0.001)

For the entire cohort, the parameters of muscle quality are as follows (M ± SD): SMD, 34.5 ± 8.9 HU; IMAT, 1118.4 ± 751.9 cm^3^; ADS, 1181.2 ± 413.4. Overall, 160 patients (20.4%) had myosteatosis, 393 patients (50.1%) had high IMAT volume values, and 362 patients (50.0%) showed low ADS. Survivors had higher muscle density and ADS in comparison to non-survivors (Table 2). There was no difference of IMAT between survivors and non-survivors. The median OS time was 11.4 months. Patients with low ADS had lower OS time in comparison to patients with high ADS (8.4 vs. 14.3 months, *p* < 0.001) (Fig. 2). Also, ADS was an independent predictor of overall survival (OS) (Table 3).

**Table 2:** Parameters of skeletal muscle quality in survivors and non survivors in the overall sample

1 year mortality			
	Survivors	Non-Survivors	*p*-value
Muscle density, M ± SD	35.4 ± 8.9	33.7 ± 9.0	0.01
IMAT volumen, M ± SD	1147 ± 749	1091 ± 755	0.30
Myosteatosis, n	67 (17.6%)	93 (23.1%)	0.06
ADS, M ± SD	1278 ± 405	1091 ± 401	< 0.01
**2 years mortality**			
Muscle density, M ± SD	35.0 ± 9.7	34.3 ± 8.7	0.39
IMAT volumen, M ± SD	1202 ± 742	1088 ± 754	0.06
Myosteatosis, %	44 (20.9%)	116 (20.2%)	0.85
ADS, M ± SD	1309 ± 429	1136 ± 399	< 0.01
**3 years mortality**			
Muscle density, M ± SD	35.6 ± 9.9	34.3 ± 8.8	0.16
IMAT volumen, M ± SD	1185 ± 744	1106 ± 753	0.29
Myosteatosis, %	21 (17.4%)	139 (21.0%)	0.36
ADS, M ± SD	1338 ± 415	1155 ± 408	< 0.01

IMAT, intramuscular adipose tissue; ADS, albumin x muscle density score

**Table 3:** Associations between overall survival and variables of the skeletal muscle quality in overall sample

Covariate	Univariable analysis			Multivariable analysis (*n* = 615)		
	HR	95% CI	*p*-value	HR	95% CI	*p*-value
Myosteatosis (vs. normal muscle density)	1.07	(0.90; 1.27)	0.46	1.01	(0.80; 1.28)	0.92
IMAT volumen (< median vs. > median)	1.02	(0.89; 1.18)	0.73	1.06	(0.89; 1.25)	0.52
ADS (< median vs. > median)	1.68	(1.45; 1.95)	< 0.01	1.55	(1.28; 1.87)	< 0.01
High Bilirubin level, (cut off = 1.2 mg/dl)	1.72	(1.49; 1.99)	< 0.01	1.48	(1.25; 1.76)	< 0.01
High TBS, (cut off = 3.37)	1.37	(1.14; 1.66)	< 0.01	1.35	(1.10; 1.65)	< 0.01
BSLC stage, (C + D vs. A + B)	3.12	(2.15; 4.54)	< 0.01	1.49	(1.22; 1.83)	< 0.01

IMAT, intramuscular adipose tissue; ADS, albumin x muscle density score

**Fig. 3:**
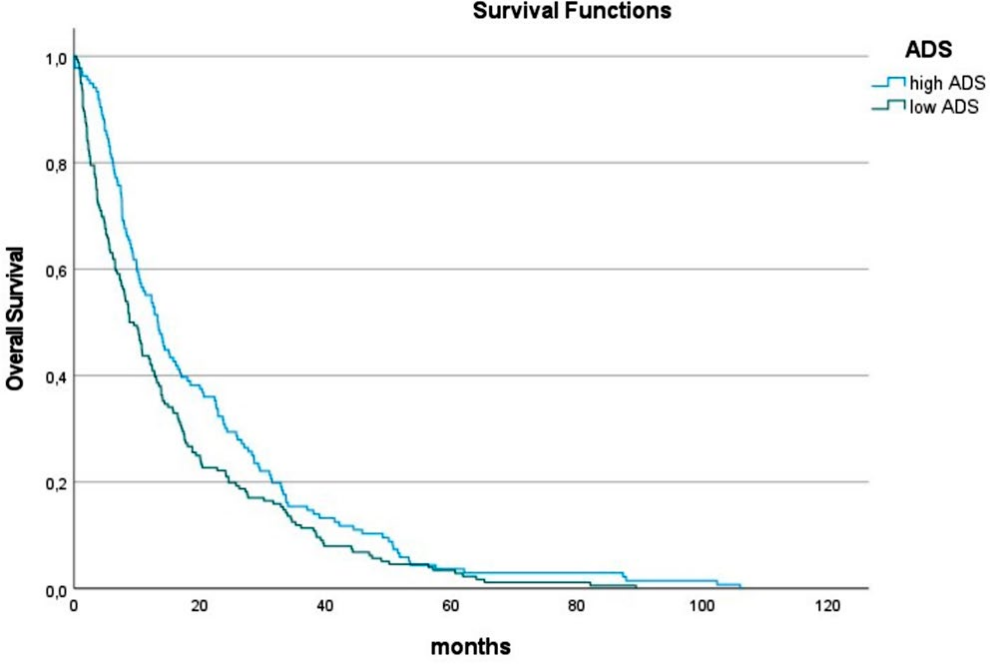
Overall survival in patients with low and normal albumin x muscle density scores (ADS) in patients with alcohol-induced HCC. Patients with low albumin x muscle density scores (ADS) had considerably lower median OS time in comparison to patients with normal ADS (13.3 vs. 15.3 months, *p* = 0.052)

In the next step, relationships between muscle quality and OS according to different etiologies were investigated. In alcohol-induced HCC, 22.7% of the patients had low skeletal muscle density, 51.8% had high IMAT values, and 54.4% showed low ADS. In this subgroup, none of the analyzed parameters of muscle quality influenced survival (Table 4; Fig. 3).

**Table 4:** Associations between overall survival and variables of the skeletal muscle quality in patients with alcohol-induced HCC

Covariate	Univariable analysis			Multivariable analysis (*n* = 260)		
	HR	95% CI	*p*-value	HR	95% CI	*p*-value
Myosteatosis (vs. normal muscle density)	1.04	0.80–1.34	0.80	1.11	0.78–1.58	0.56
IMAT volumen (< median vs. > median)	1.03	0.83–1.28	0.76	1.07	0.83–1.38	0.62
ADS (< median vs. > median)	1.32	1.06–1.66	0.02	1.14	0.85–1.54	0.37
High Bilirubin level, (cut off = 1.2 mg/dl)	1.56	1.25–1.95	< 0.01	1.41	1.08–1.83	0.01
High TBS, (cut off = 3.37)	1.30	0.98–1.73	0.07	1.38	1.02–1.87	0.04
BSLC stage, (C + D vs. A + B)	2.42	1.83–3.19	< 0.01	1.97	1.41–2.74	< 0.01

IMAT, intramuscular adipose tissue; ADS, albumin x muscle density score

**Fig. 4:**
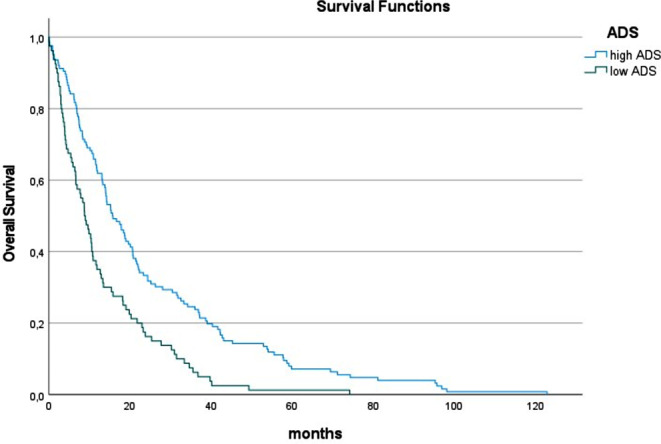
Overall survival in patients with low and normal albumin x muscle density scores (ADS) in patients with viral-induced HCC. Patients with low ADS had shorter median OS time than patients with high ADS (8.8 vs. 15.7 months, *p* < 0.001)

In viral-induced HCC, 12.4% of the patients had skeletal muscle density, 38.7% had high IMAT values, and 39.0% showed low ADS. In this subgroup, low ADS was a strong and independent predictor of OS (Table 5). Patients with low ADS had shorter OS time than patients with high ADS (8.8 vs. 15.7 months, *p* < 0.001) (Fig. 4).

**Table 5:** Associations between overall survival and variables of the skeletal muscle quality in patients with viral-induced HCC

Covariate	Univariable analysis			Multivariable analysis (*n* = 179)		
	HR	95% CI	*p*-value	HR	95% CI	*p*-value
Myosteatosis (vs. normal muscle density)	0.95	0.64–1.41	0.80	0.78	0.45–1.37	0.39
IMAT volumen (< median vs. > median)	1.13	0.86–1.48	0.38	1.18	0.86–1.64	0.31
ADS (< median vs. > median)	1.89	1.41–2.53	< 0.01	2.04	1.38–3.03	< 0.01
High Bilirubin level, (cut off = 1.2 mg/dl)	1.66	1.27–2.17	< 0.01	1.44	1.04–2.00	0.03
High TBS, (cut off = 3.37)	1.54	1.11–2.15	0.01	1.38	0.96–1.98	0.08
BSLC stage, (C + D vs. A + B)	1.60	1.16–2.21	0.01	1.30	0.88–1.93	0.19

IMAT, intramuscular adipose tissue; ADS, albumin x muscle density score

**Fig. 5:**
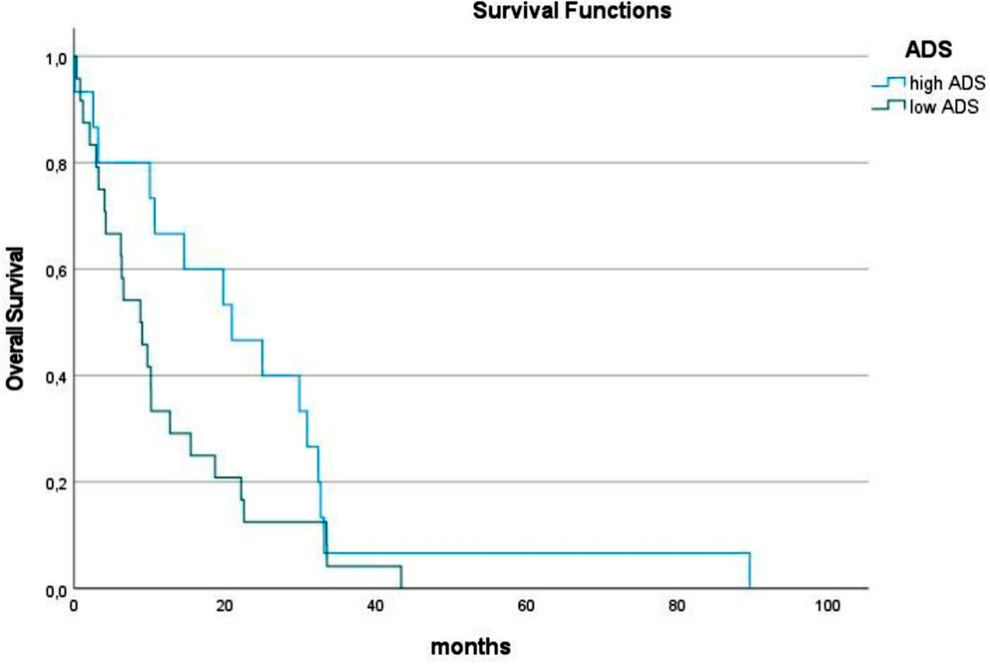
Overall survival in patients with low and normal albumin x muscle density scores (ADS) in patients with NASH-induced HCC. Patients with low ADS have lower median survival time than patients with high ADS, 12.0 vs. 23.7 months, respectively (*p* = 0.12)

Finally, in NASH-induced HCC, 23.8% of the patients had skeletal muscle density, 47.6% had high IMAT values, and 61.5% showed low ADS. None of the analyzed parameters of muscle quality influenced survival (Table 6; Fig. 5).

**Table 6:** Associations between overall survival and variables of the skeletal muscle quality in patients with NASH-induced HCC

Covariate	Univariable analysis			Multivariable analysis (*n* = 38)		
	HR	95% CI	*p*-value	HR	95% CI	*p*-value
Myosteatosis (vs. normal muscle density)	1.33	0.64–2.78	0.44	0.94	0.35–2.52	0.90
IMAT volumen (< median vs. > median)	0.66	0.35–1.25	0.20	0.32	0.14–0.76	0.01
ADS (< median vs. > median)	1.70	0.87–3.33	0.12	1.27	0.56–2.91	0.57
High Bilirubin level, (cut off = 1.2 mg/dl)	2.32	1.21–4.42	0.01	3.59	1.57–8.24	< 0.01
High TBS, (cut off = 3.37)	1.29	0.50–3.35	0.60	0.92	0.29–2.90	0.88
BSLC stage, (C + D vs. A + B)	1.34	0.66–2.71	0.42	1.59	0.63–4.02	0.32

IMAT, intramuscular adipose tissue; ADS, albumin x muscle density score

## Discussion

The present study demonstrated that low muscle quality represented by myosteatosis, high IMAT, and low ADS are frequent in patients with advanced HCC. More importantly, low ADS is an independent predictor of poor survival in HCC. High IMAT and myosteatosis, however, did not influence survival in our sample.

According to the literature, in HCC, parameters of the skeletal muscle quality are significant predictors of overall survival (Aleixo et al. 2020; Fujiwara et al. 2015). However, most previous studies analyzed the predictive role of the musculature in patients undergoing treatment with different kinase inhibitors or hepatectomy (Aleixo et al. 2020). Only two reports studied previously the role of skeletal muscle quality in patients with HCC undergoing treatment with TACE (Bannangkoon et al. 2023; Masetti et al. 2022). Furthermore, the reported data are controversial. For instance, in the study of Bannangkoon et al., patients with myosteatosis had shorter overall survival than those without myosteatosis (15.9 vs. 27.1 months, *P* < 0.001) (Bannangkoon et al. 2023). However, according to Masetti et al., myosteatosis was not associated with complications or survival in HCC patients undergoing transarterial embolization (Masetti et al. 2022). In summary, previous studies analyzed only myosteatosis or low skeletal muscle density as parameters of low muscle quality. In the present work, we investigated the prognostic role of different values of muscle quality. Recently, a study proposed a combined value of muscle density and albumin level in patients with non-metastatic colorectal cancers (Kim et al. 2023). The authors found that this score was an independent predictor of survival, with superior prognostic value compared to skeletal muscle index, skeletal muscle density or albumin alone (Kim et al. 2023).

Serum albumin is a well-known marker of systemic inflammation and nutritional status (Gupta et al. 2010). Albumin reflects the severity of liver functional impairment (Gupta et al. 2010). Hepatic function is a key prognostic marker in patients with HCC and central to patient selection for TACE (Galle et al. 2018).

Relationships between skeletal muscle quality and OS in patients with advanced HCC may be caused by several factors. In first instance, low muscle density and ADS are both parameters reflecting malnutrition and low serum albumin level. Furthermore, low muscle quality may be associated with altered endocrine function of the skeletal musculature. It is known that skeletal muscles synthesize and secret several peptides (myokines) with anticancer effects (Park et al. 2023). We hypothesize that low muscle quality reflected by low ADS may be associated with a reduction of myokine synthesis and secretion. This may cause a reduction of circulating and intratumoral immune cells.

Interestingly, IMAT values did not influence survival in our sample. This finding indicates that fatty infiltration of the perimuscular space reflected by IMAT may not affect the endocrine and antitumoral effects of the skeletal musculature in contrast to intracellular muscle degeneration reflected by low muscle density.

Our study has further important findings. We identified that patients with alcohol-induced HCC and NASH had lower muscle quality than patients with viral-induced HCC. This finding is not unusual and may be related to the known malnutrition in patients with alcoholism. Patients with NASH show systemic metabolic disorders with deposits of adipose tissue within several organs. ADS affected OS in patients with viral-induced HCC but not in alcohol-induced and NASH induced tumors. This phenomenon may be explained by the fact that the frequency of low ADS in patients with alcohol-induced and NASH-induced HCCs is significantly higher than in patients with viral-induced HCC. This finding is of great clinical importance and it indicates that the etiology of HCC should be taken into account when using the skeletal muscle quality to determine treatment strategy. Also, this phenomenon may explain conflicting results of the previous studies.

The identified results are highly important for clinical practice. Low muscle quality is a modifiable factor. According to the literature, exercise and an additional nutritional support with vitamins and proteins can improve muscle quality in tumor patients (Marcantei et al. 2024). Therefore, check for low muscle quality, especially for low ADS, and development of supportive regimes may be of benefit for patient with advanced HCC.

Overall, our data underlines the importance of the quantitative analysis of body composition in patients with HCC and supports the results of previous investigations (Xiong et al. 2023; Surov et al. 2024).

Some limitations of the present study are to address. Firstly, this is a retrospective analysis. Secondly, we excluded patients with missing baseline abdominal CT scan that might lead to selection bias. Thirdly, the NASH cohort in our sample is small and, therefore, our results about the role of muscle quality in this subgroup do not allow drawing definitive conclusions.

In conclusion, low ADS is an independent predictor of worse OS in patients with viral-induced HCC undergoing treatment with TACE. In alcohol-induced and NASH-induced HCCs, parameters of muscle quality do not influence OS.

## Electronic supplementary material

Below is the link to the electronic supplementary material.

Supplementary Material 1

## Data Availability

No datasets were generated or analysed during the current study.

## Abbreviations

ADS: Albumin-density score
BCLC: Barcelona Clinic Liver Cancer classification
CT: Computed tomography
TBS: Tumor burden score
EASL: European Association for the Study of Liver Disease
HCC: Hepatocellular carcinoma
HU: Hounsfield unit
IMAT: Intramuscular adipose tissue
OS: Overall survival
SMD: Skeletal muscle density
TACE: Transarterial chemoembolization
