# Supplementary material for: Albumin-muscle density score predicts overall survival in patients with hepatocellular cancer undergoing treatment with transarterial chemoembolization
Source: J Cancer Res Clin Oncol. 2024 Nov 30;150(12):515. doi: 10.1007/s00432-024-06043-3 (PMC11608168; doi:10.1007/s00432-024-06043-3)
Supplement: Supplementary file 1 — Supplementary Material 1 [file 432_2024_6043_MOESM1_ESM.docx]

**Suppl table 1. Centers, patients, and imaging parameters**

| **Centers** | **Number of involved cases** | **CT scanners (Manufacturer, Headquater)** | **Slice thickness, mm** |
| --- | --- | --- | --- |
| Mainz | 265 | iCT and Brilliance 64 (Philips, Eindhoven, the Netherlands) | Reconstructing images in the axial orientation, as well as sagittal and coronal views, with slice thicknesses of 1 mm, 3 mm, and 5 mm. |
| Freiburg | 217 | Definition Flash, Force or Definition AS (Siemens Healthineers, Forchheim, Germany) | Reconstructions were performed at 1 mm and 3 mm slice thickness in multiplanar directions. |
| Berlin | 113 | Revolution HD (GE Healthcare. Chicago, Illionois, USA) | Reconstruction was performed with a slice thickness of 1 mm and 5 mm in axial and coronal orientations. |
| Heidelberg | 67 | Somatom Definition Flash, Siemens Healthineers (Forchheim, Germany), Spectral CT scanner 7500 (Philips, Eindhoven, the Netherlands) | Image reconstruction was performed with a slice thickness of 1 mm and 5 mm in axial and coronal orientations. |
| Cologne | 47 | IQon Spectral CT (Philips, Eindhoven, the Netherlands) | Image reconstruction was performed with a slice thickness of 1 mm and 5 mm in axial and coronal orientations. |
| Essen | 45 | SOMATOM Definition Flash, SOMATOM Force, or SOMATOM Definition AS (Siemens Healthineers, Forchheim, Germany) | Image reconstruction was performed with a slice thickness of 1 mm and 5 mm in axial and coronal orientations. |
